# Cardiac output estimated from an uncalibrated radial blood pressure waveform: validation in an *in-silico*-generated population

**DOI:** 10.3389/fbioe.2023.1199726

**Published:** 2023-05-30

**Authors:** Vasiliki Bikia, Georgios Rovas, Nikolaos Stergiopulos

**Affiliations:** Laboratory of Hemodynamics and Cardiovascular Technology, Institute of Bioengineering, Swiss Federal Institute of Technology, Lausanne, Switzerland

**Keywords:** arterial pulse wave, vascular age, non-invasive monitoring, neural networks, cuff-free

## Abstract

**Background:** Cardiac output is essential for patient management in critically ill patients. The state-of-the-art for cardiac output monitoring bears limitations that pertain to the invasive nature of the method, high costs, and associated complications. Hence, the determination of cardiac output in a non-invasive, accurate, and reliable way remains an unmet need. The advent of wearable technologies has directed research towards the exploitation of wearable-sensed data to improve hemodynamical monitoring.

**Methods:** We developed an artificial neural networks (ANN)-enabled modelling approach to estimate cardiac output from radial blood pressure waveform. *In silico* data including a variety of arterial pulse waves and cardiovascular parameters from 3,818 virtual subjects were used for the analysis. Of particular interest was to investigate whether the uncalibrated, namely, normalized between 0 and 1, radial blood pressure waveform contains sufficient information to derive cardiac output accurately in an *in silico* population. Specifically, a training/testing pipeline was adopted for the development of two artificial neural networks models using as input: the calibrated radial blood pressure waveform (ANN_calradBP_), or the uncalibrated radial blood pressure waveform (ANN_uncalradBP_).

**Results:** Artificial neural networks models provided precise cardiac output estimations across the extensive range of cardiovascular profiles, with accuracy being higher for the ANN_calradBP_. Pearson’s correlation coefficient and limits of agreement were found to be equal to [0.98 and (−0.44, 0.53) L/min] and [0.95 and (−0.84, 0.73) L/min] for ANN_calradBP_ and ANN_uncalradBP_, respectively. The method’s sensitivity to major cardiovascular parameters, such as heart rate, aortic blood pressure, and total arterial compliance was evaluated.

**Discussion:** The study findings indicate that the uncalibrated radial blood pressure waveform provides sample information for accurately deriving cardiac output in an *in silico* population of virtual subjects. Validation of our results using *in vivo* human data will verify the clinical utility of the proposed model, while it will enable research applications for the integration of the model in wearable sensing systems, such as smartwatches or other consumer devices.

## Introduction

Cardiac output (CO) is defined as the volume of blood expelled by the left ventricle per unit time. Critically ill patients generally have abnormal oxygen demands as a result of the underlying diseases. Thus, CO monitoring is essential for patient management in the operating room and the intensive care unit (ICU) (Berkenstadt et al., 2001; Lees et al., 2009). Direct methods for measuring CO include invasive approaches, such as the Fick method and the thermodilution method. Alternative approaches, such as pulse contour analysis (Udy et al., 2012), have been put forth as less invasive methods. However, pulse contour analysis necessitates the placement of a pressure catheter at an arterial site (Jansen et al., 2001; Udy et al., 2012; Ganter et al., 2016).

On the other hand, non-invasive methods for CO have been introduced in order to overcome the complications and potential risk of the invasive and minimally invasive techniques. Some non-invasive methods are based on pulse wave analysis from the cross-sectional area and blood velocity data (Segers et al., 2007) or directly from MRI-derived aortic flow-time signals (Hickson et al., 2010). Doppler ultrasound and MRI, while completely non-invasive and reasonably accurate, require the allocation of expensive resources. It is of interest to mention that a previous study (Zócalo et al., 2021) investigated the impact of sex, age, heart rate, and anthropometric characteristics on the estimation of stroke volume, CO, and cardiac index in a large cohort of 1,449 healthy subjects covering a wide range of age values (3–88 years). CO was non-invasively obtained based on pulse contour analysis (PCA) on brachial BP acquired using the Mobil-O-Graph device (Mobil-O-Graph; Germany). Importantly, the study demonstrated that gender, age, heart rate, and body surface area are independent factors that explain PCA-derived CO values, suggesting that they should be taken into account in CO monitoring applications. Moreover, impedance cardiography (ICG) provides another rather clinically relevant alternative to monitor stroke volume and CO, allowing for the assessment of these parameters during both stress conditions and at rest (Liu et al., 2021). Yet, none of the aforementioned methods are practical for continuous bedside monitoring of a patient’s CO or routine examination. As a result, the determination of CO in a non-invasive, accurate, and reliable way remains an unmet need.

Recent advances in measuring sensors have spurred the development of a gamut of methods to calculate CO from arterial blood pressure (BP) signals, with many of them being commercially available. The main aspects that have encouraged this approach include: (i) the fact that arterial BP can be acquired in a relatively easy, non-invasive (or minimally invasive), and cost-effective manner; (ii) arterial BP is measured in clinical settings such as ICUs on a routine basis; and (iii) the arterial BP is measured continuously, allowing for continuous CO estimates. In addition, the advent of wearable technologies has enabled research efforts towards the exploitation of wearable-sensed data to improve hemodynamical monitoring. Especially, smartwatches and fitness bands can provide access to peripheral arterial pulse waves, which could be afterwards further analyzed in order to provide major hemodynamic parameters, such as arterial stiffness, cardiac output, etc.

In this study, we introduced a novel machine learning-enabled method to estimate CO from radial BP waveform. Given that simultaneous invasive radial BP and CO data are typically difficult to acquire *in vivo*, we leveraged a previously generated *in silico* dataset simulating 3,818 virtual subjects (Bikia et al., 2021). A training/testing pipeline using artificial neural networks (ANN) was adopted for the development of two ANN models using as an input vector: (i) the calibrated radial BP waveform, or (ii) the uncalibrated radial BP waveform. The performance of the resulted predictive models was evaluated by comparing the model-derived values with the reference CO data.

## Materials and methods

### *In silico* population

In this study, we used an *in silico* dataset from our previously published work (Bikia et al., 2021). The data generation relied on a previously developed, clinically validated one-dimensional cardiovascular computer simulator (Reymond et al., 2009) and intended to emulate the content of various hemodynamical profiles. The cardiovascular model (Reymond et al., 2009) ran using different combinations of input model parameters based on publicly available literature data which were varied using a random Gaussian distribution. The parameters of arterial distensibility, terminal compliance, and peripheral resistance were altered in order to achieve the specific value in the selected ranges. Furthermore, the length, inlet diameter, and outlet diameter of every arterial segment was modified to simulate different body types by adapting the length and the diameter of all arterial vessels. The reader is referred to the original publication (Bikia et al., 2021) for the detailed description of the data design and generation.

The simulated radial BP waveform was derived from the virtual left radial artery. In addition, *in silico* BP values, such as the mean arterial pressure, systolic and diastolic BP, and pulse pressure at the aortic root became available from the model-simulated aortic BP waveform. CO was calculated as the product of heart rate and stroke volume, which was calculated from the area under the curve of the aortic blood flow rate. Values of total arterial compliance were derived analytically by summing the incremental volume compliance of all arterial segments. The data were organized in input-output pairs for every virtual subject, namely, the radial BP waveform and the respective CO value were assigned to every virtual subject.

### Data analysis

**FIGURE 1:**
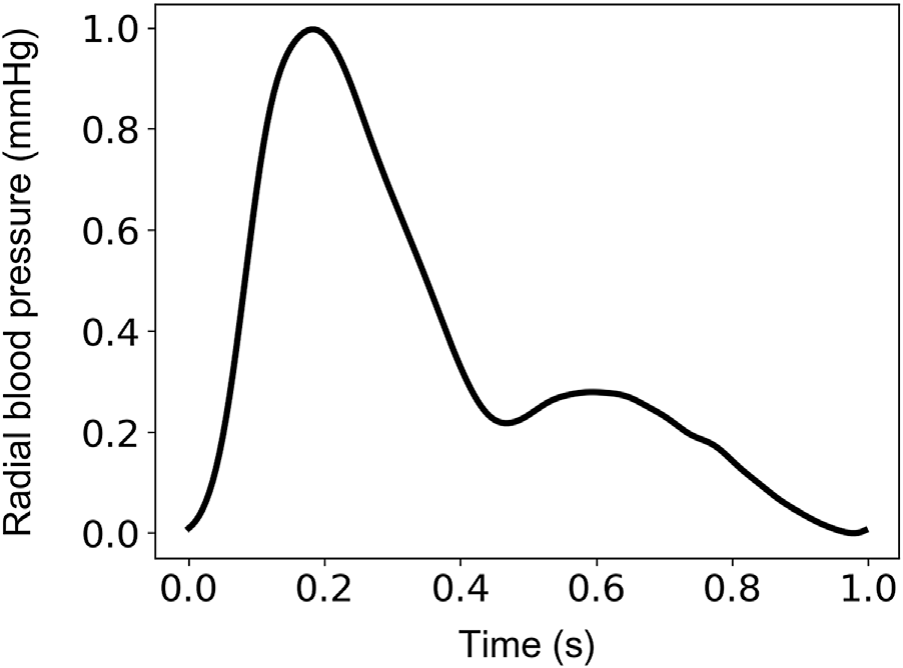
Indicative uncalibrated (normalized between 0 and 1) radial blood pressure waveform.

The input-output sets were subsequently divided into train, validation, and test sets. The train/validation/test split was set to be 60% (2,290 cases)/20% (764 cases)/20% (764 cases). The sampling frequency was set to 128 Hz. This selection allowed us to ensure a sampling frequency higher than the 100-Hz threshold suggested for the pulse wave velocity techniques (Gaddum et al., 2013) (which require substantially high temporal resolution). This value was considered as a fair trade-off between computational time and high signal fidelity. Normalization of the radial BP waveforms was performed between 0 and 1 using the linear scaling formulation. An indicative example of an uncalibrated radial BP waveform normalized between 0 and 1 is shown in Figure 1.

### Artificial neural networks model

We used artificial neural networks (ANN) to estimate the target variable of interest, namely, CO. For the ANN, a fixed one-hidden layer structure was selected and the “Adam” optimizer was used (Kingma and Jimmy, 2017). A training/testing pipeline was adopted for the development of two ANN models using as an input vector: (i) the calibrated radial BP waveform (ANN_calradBP_), or (ii) the uncalibrated radial BP waveform (ANN_uncalradBP_). Subsequently, the performance of the trained predictive models was evaluated by comparing the model-derived values with the reference CO data.

To mitigate overfitting and to increase the generalization capacity, machine learning models should be trained for optimal hyperparameter values. For the ANN, the batch size (defines the number of samples that will be propagated through the network) was set to be equal to 64, whereas the number of epochs was optimized. The number of epochs defines the number of times that the learning algorithm works through the entire training data set. For selecting the optimal value of epochs, we computed the training loss and the validation loss for various values of epochs. Loss values (using the mean squared error method) were monitored by an early-stopping call-back function. When an increment is observed in the loss values, training comes to an halt and the respective value of epoch indicates the optimal selection. Description of the ANN-based models and the selected number of epochs that was computed are presented in Table 1. Subsequently, the test set was fed into the trained models to predict CO and the precision was evaluated. The training/testing pipeline as well as the pre-analyses and post-analyses were implemented using the Scikit-learn library (Pedregosa et al., 2011) in a Python programming environment. The Pandas and Numpy packages were used (Oliphant, 2006; McKinney, 2010).

**TABLE 1:** Description of the artificial neural networks-based models.

Model	Input	Batch size	Selected number of epochs
ANN_calradBP_	Calibrated radial blood pressure waveform	64	61
ANN_uncalradBP_	Uncalibrated radial blood pressure waveform	64	71

**FIGURE 2:**
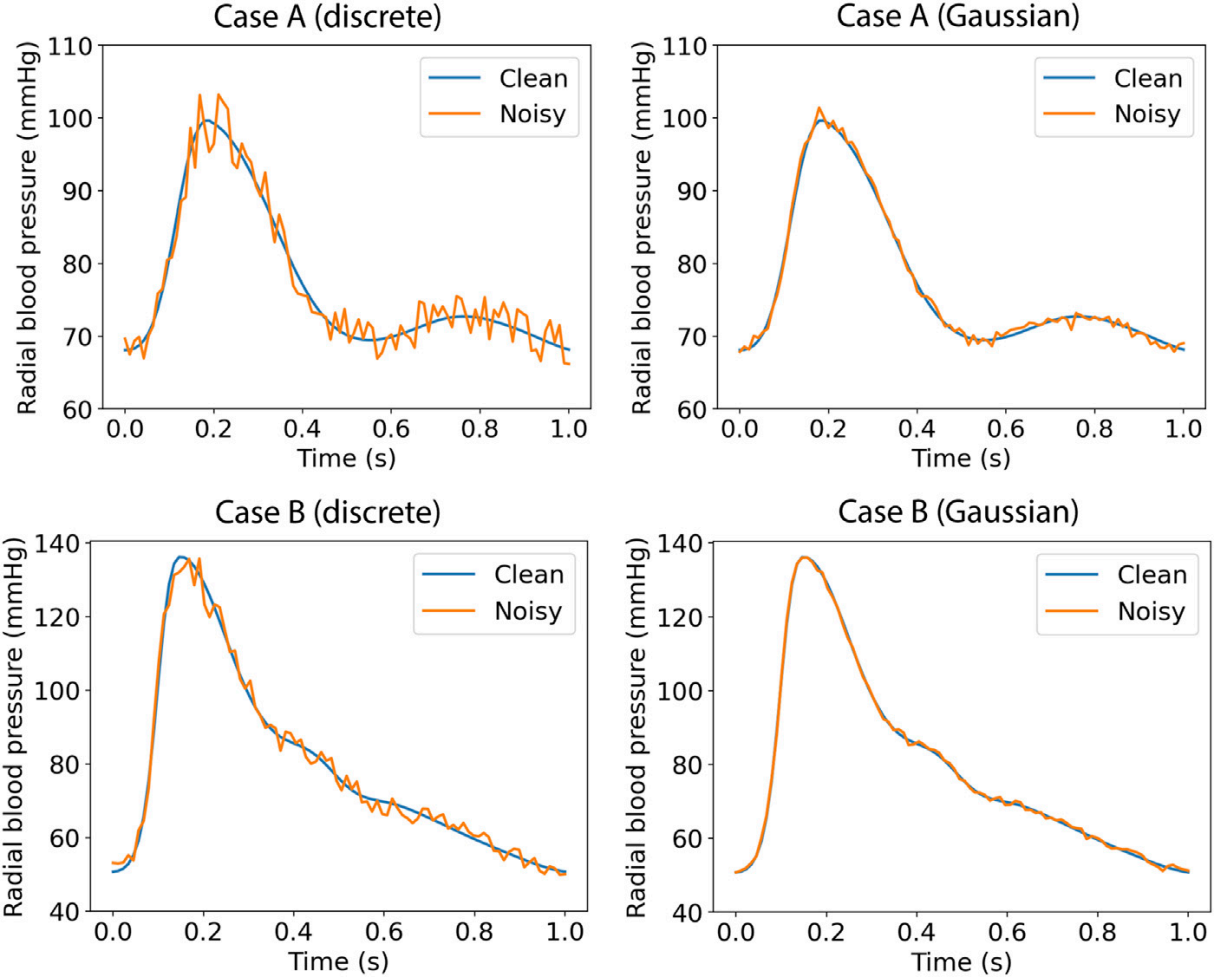
Radial blood pressure data with added artificial random noise.

*In silico* data cannot simulate different aspects that usually occur with real data registries (e.g., imperfection of signals, over-damping, etc.). In order to assess the performance of the proposed method on input signals with errors or other imperfections, we tested the ANN models (using the same model configuration) after artificially adding random noise. Firstly, we selected a more brute-force scenario where the error for each pressure data point was randomly drawn from the range of ±5%. Each pressure data point was multiplied with a random noise factor; for instance, for a randomly selected error of −3%, the respective variable value was multiplied with a noise factor equal to 0.97. Secondly, to simulate more realistic measurement errors, we added artificial random noise assuming a Gaussian distribution with *μ* = 0.7 mmHg, and SD = 1 mmHg. Indicative examples of the noisy data are shown in Figure 2.

### Statistical analysis

All data are presented as mean and standard deviation (SD). The statistical analysis was performed in Python (Python Software Foundation, Python Language Reference, version 3.6.8, available at http://www.python.org). The correlation and precision between the estimations and the reference data were evaluated using the Pearson’s correlation coefficient (*r*), and the normalized root mean square error (nRMSE). The computed nRMSE was based on the difference between the minimum and maximum values of the dependent variable (y) and was computed as RMSE/(y_max_–y_min_). Bias and limits of agreement (LoA) (where the 95% of errors are expected to lie) were calculated using the Bland-Altman analysis (Bland and Altman, 1986). Linear least-squares regression was performed for the estimated and reference data. The slope and the intercept of the regression line were reported. Two-sided *p*-values for hypothesis tests were calculated using Wald Tests with t-distribution of the test statistic. The null hypothesis was that the slope is zero. A *p* < 0.05 was considered statistically significant.

## Results

**FIGURE 3:**
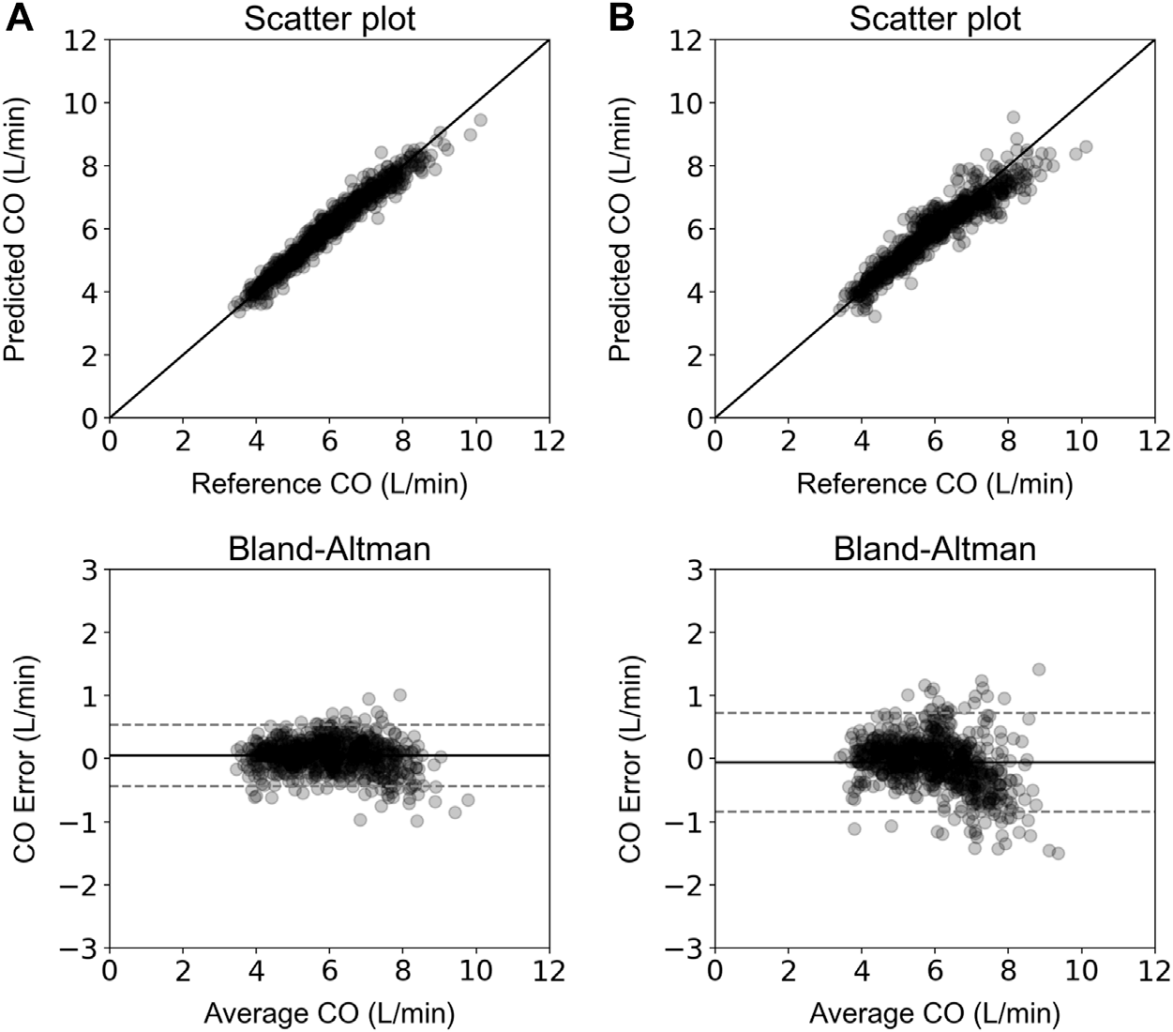
Scatter plot and Bland-Altman analysis between the estimated and reference CO values for the ANN_calradBP_ **(A)** and ANN_uncalradBP_ **(B)**. The solid line of the scatterplots represents equality. In Bland–Altman plots, the solid black line represents the bias and the two horizontal dashed lines define the limits of agreement (LoA), within which 95% of errors are expected to lie.

Table 2 aggregates the cardiovascular parameters of the *in silico* data (*n* = 3,818). Comparison between the model-derived predictions and the reference data was performed. Accuracy was reported to be increased for the ANN model that used the calibrated radial BP waveform as an input (*r* = 0.98). In both models, LoA were narrow and biases were found to be close to zero. The scatter plots and the Bland–Altman plots of the estimated CO for the two ANN models against the ground truth are shown in Figure 3. For the ANN models using the noisy data as input, the accuracy was lower with increasing level of assumed noise. The ANN_calradBP_ was found to be more robust to the addition of random noise in comparison to the ANN_uncalradBP_. Overall, correlation values between the estimated and reference CO data remained at satisfactory levels (≥0.77). In the case of ANN_calradBP_, nRMSE values were low (≤6 %). The nRMSE was essentially increased in ANN_uncalradBP_, especially when discrete random noise was added to the signals (nRMSE was doubled). All regression metrics for the agreement, precision, and bias are presented in Table 3.

**TABLE 2:** Description of the cardiovascular parameters of the *in silico* data.

Parameter	*In silico* population *n* = 3,818		
	Min	Max	Mean ± SD
Aortic systolic BP (mmHg)	77	187	123 ± 24
Aortic diastolic BP (mmHg)	43	128	80 ± 21
Aortic pulse pressure (mmHg)	11	106	42 ± 19
Mean arterial pressure (mmHg)	66	155	101 ± 21
Radial systolic BP (mmHg)	85	193	133 ± 23
Radial diastolic BP (mmHg)	37	119	73 ± 21
Radial pulse pressure (mmHg)	21	124	60 ± 22
Heart cycle (s)	0.6	1	0.7 ± 0.1
Total peripheral resistance (mmHg·s/mL)	0.6	1.4	1 ± 0.2
Total arterial compliance (mL/mmHg)	0.3	2.9	1.1 ± 0.5
Cardiac output (L/min)	3.3	10.5	6 ± 1.2

**TABLE 3:** Accuracy, agreement, and correlation between ANN-predicted and reference CO data.

Model	*r*	MAE (L/min)	nRMSE (%)	Bias (LoA) (L/min)	Reference CO (L/min)	Estimated CO (L/min)	Slope/Intercept
ANN_calradBP_ (no noise)	0.98	0.19	3.7	0.05 (−0.44, 0.53)	6 ± 1.2	6 ± 1.2	0.95 (*p* < 0.0001)/0.32 L/min
ANN_calradBP_ (discrete)	0.95	0.28	5.7	−0.03 (−0.74, 0.68)	5.8 ± 1.2	5.8 ± 1.2	0.95 (*p* < 0.0001)/0.25 L/min
ANN_calradBP_ (Gaussian)	0.97	0.34	6	−0.28 (−0.84, 0.28)	6 ± 1.2	5.7 ± 1.1	0.91 (*p* < 0.0001)/0.26 L/min
ANN_uncalradBP_ (no noise)	0.95	0.3	6	−0.06 (−0.84, 0.73)	6 ± 1.2	5.9 ± 1.1	0.85 (*p* < 0.0001)/0.84 L/min
ANN_uncalradBP_ (discrete)	0.77	0.69	12.3	0.13 (−1.56, 1.83)	6.1 ± 1.3	6.2 ± 1.3	0.79 (*p* < 0.0001)/1.39 L/min
ANN_uncalradBP_ (Gaussian)	0.88	0.45	8.6	−0.02 (−1.17, 1.12)	6 ± 1.2	5.9 ± 1.1	0.78 (*p* < 0.0001)/1.29 L/min

*r*, Pearson’s correlation coefficient; MAE, mean absolute error; nRMSE, normalized root mean square error; LoA, limits of agreement.

**FIGURE 4:**
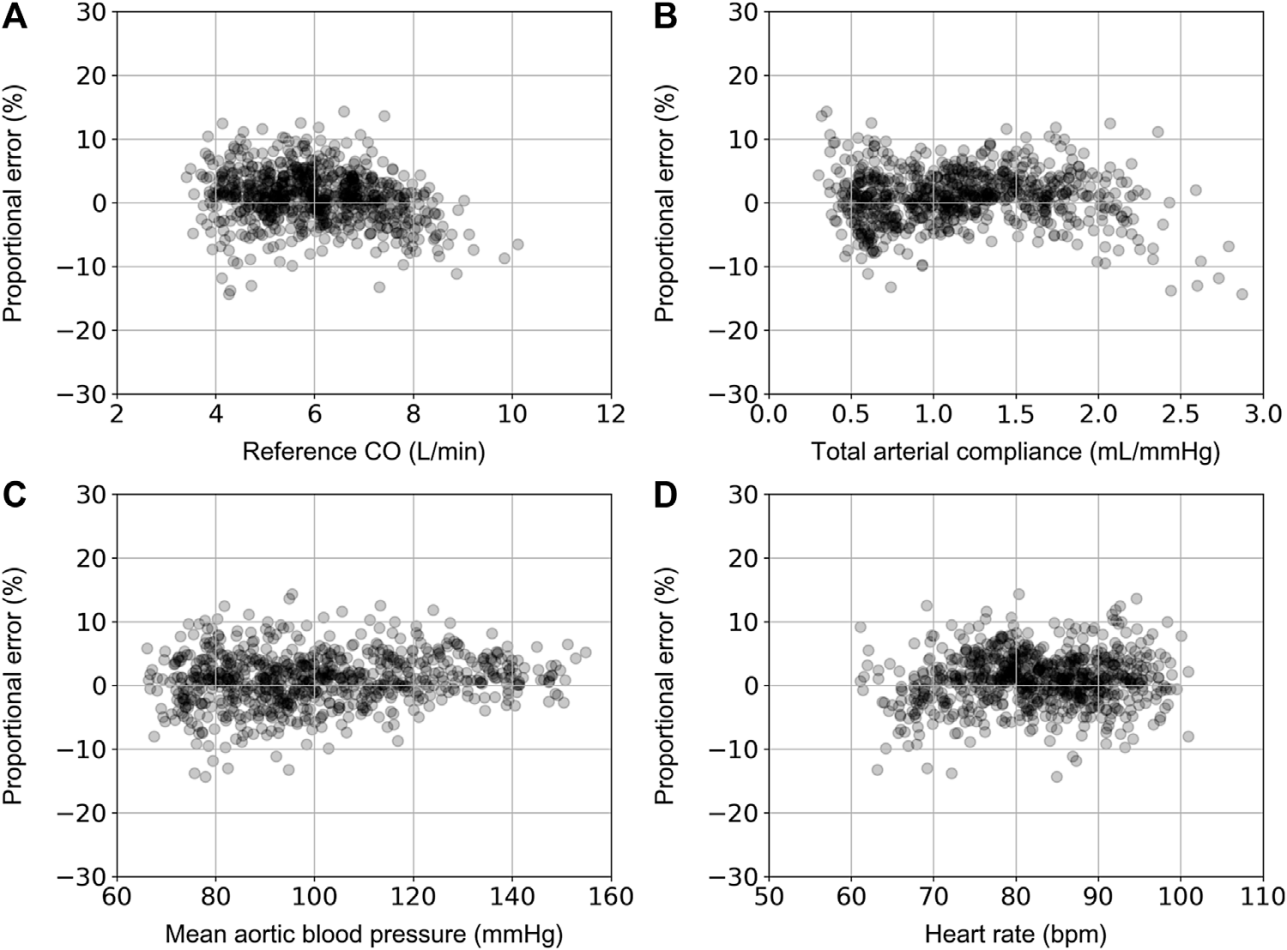
Comparison of proportional error, calculated as CO_predicted_ – CO_reference_)/CO_reference_, with reference CO **(A)**, total arterial compliance **(B)**, mean aortic blood pressure **(C)**, and heart rate **(D)**.

**FIGURE 5:**
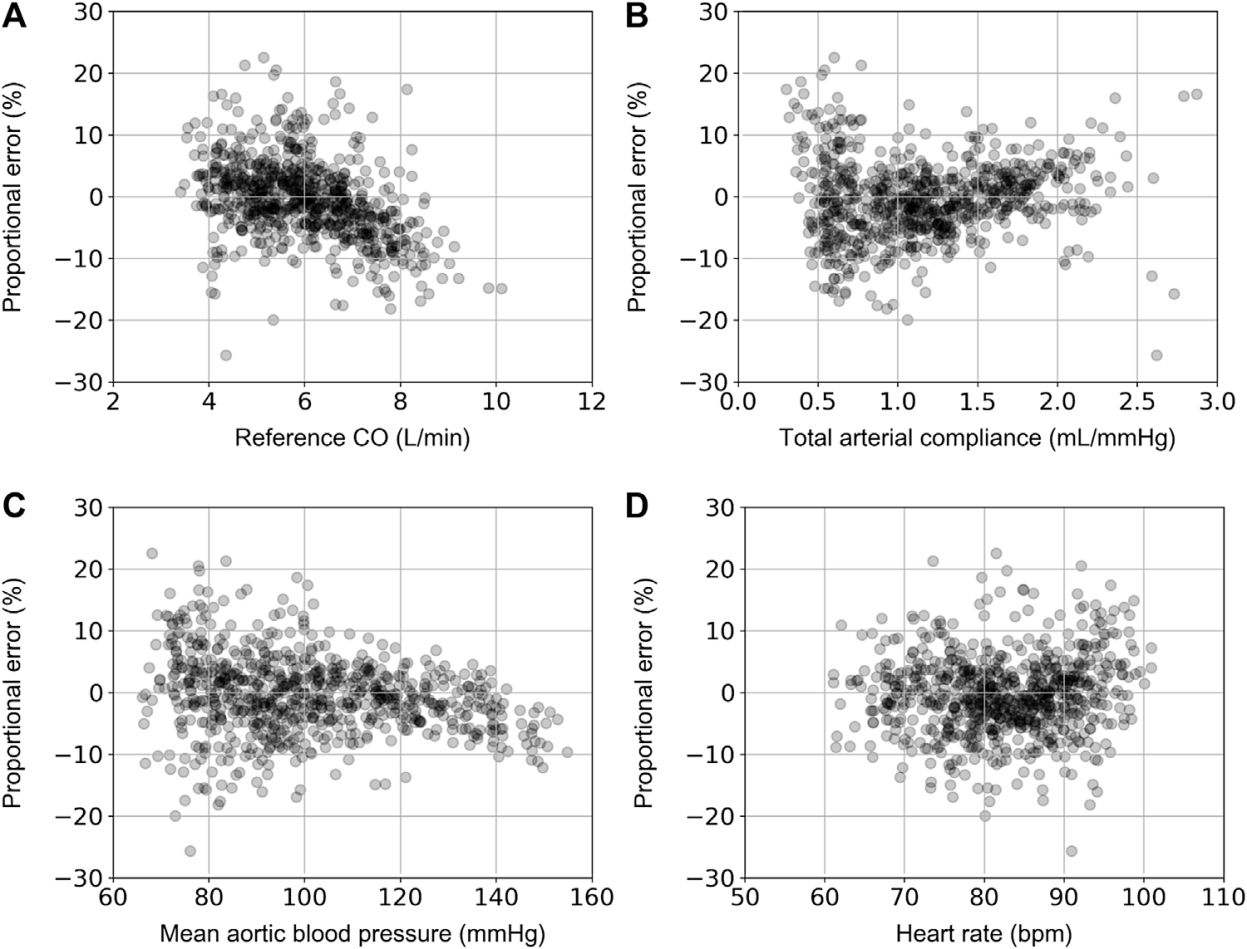
Comparison of proportional error, calculated as CO_predicted_ – CO_reference_)/CO_reference_, with reference CO **(A)**, total arterial compliance **(B)**, mean aortic blood pressure **(C)**, and heart rate **(D)**.

For the ANN_calradBP_, the proportional error (PE) with respect to the reference CO values is shown in Figure 4. The PE was calculated as (CO_predicted_ – CO_reference_)/CO_reference_.The maximum and minimum PE values were reported to be 14.4% and −14.3%, respectively. In addition, the distribution of PE was found to be equal to 0.9% ± 4.1%. The respective values for the ANN_uncalradBP_ were the following: PE_max_ = 22.6%, PE_min_ = −25.6%, PE_mean_ = −0.5%, and PE_SD_ = 6.3% (Figure 5). The PE demonstrated an increasing trend with increasing CO values. No correlation was reported for the PE resulted from both ANN_calradBP_ and ANN_uncalradBP_ models with respect to heart rate, total arterial compliance, and mean aortic blood pressure (Figures 3, 4).

## Discussion

This article introduced an ANN-enabled method to estimate CO from the radial BP waveform. In particular, we investigated the concept of deriving CO from, firstly, the calibrated radial BP waveform and, secondly, the morphology of the raw uncalibrated radial BP wave using an in-silico-generated virtual population of various hemodynamical profiles. The findings indicated that CO can be precisely predicted by exploiting the radial (peripheral) BP pulse wave. This method relies on the raw information hidden in the radial pulse wave that can be deciphered via the predictive capacity of neural networks with relatively simple structure. Ultimately, potential use of such methods could include the expansion and integration of a prediction algorithm to estimate CO using wearable sensing technologies (such as smartwatches or fitness trackers), while eliminating the need for complex and expensive echocardiographic or MRI procedures.

Cardiac output (CO) is a key parameter in assessing circulatory function. Currently in clinical practice, the gold standard for CO measurement is thermodilution CO (TCO), which involves the insertion of a catheter into the pulmonary artery. Conducted primarily in ICUs, TCO is usually measured intermittently, is very invasive, and may cause severe complications. It would be a tremendous asset to healthcare if one could determine CO accurately, reliably, and continuously using less invasive, indirect methods. Over the last decades, numerous methodologies have been suggested and developed to estimate CO using peripheral arterial BP data obtained either minimally invasively or non-invasively (Sun et al., 2005; Litton and Morgan, 2012; Bikia et al., 2020a; Bikia et al., 2020b; Bikia, 2021; Saugel et al., 2021). Some of these estimators rely on elaborate models of the heart and vasculature while others use artificial intelligence methods such as pattern matching and classification trees. The majority of the published estimators has not been extensively evaluated with a large set of clinical arterial blood pressure data, hence their performance may need to be re-evaluated. An additional issue to be considered with regards to the validation of CO estimation techniques pertains to the establishment of universal, well-defined thresholds of acceptable accuracy. In contrast to the BP monitors, CO data from meta-analyses studies are limited, with only few and not up-to-date works trying to propose threshold of error values that could allow a newly introduced method to be considered as reliable, repeatable, and accurate (Critchley and Critchley, 1999; Critchley et al., 2014).

Typically, recording the calibrated radial BP waveform requires calibration using a conventional cuff procedure. At large, cuff-based devices have been widely used for non-invasive BP assessment and their utility is critical for several medical conditions (Tzourio et al., 2017). Nonetheless, there are various limitations in the use of methods relying on cuff-based BP measurement, including: i) low accuracy (Harris et al., 2016), ii) the measurement is usually intermittent and does not capture all BP changes occurring throughout the recording window, ii) and existing devices are bulky, not portable and thus not practical for daily use (O’Brien et al., 2013; Parati et al., 2014).

Many cuffless BP estimation methods have been proposed to overcome these limitations, enabling continuous BP monitoring (Kim et al., 2012; Kim et al., 2007; Baek, 2009), but not CO derivation. The tremendous majority of such methods is based on the pulse transit time (PTT) principle. PTT is the pulse wave propagation time, which represents the time required for the wave to travel between two arterial sites within the same cardiac cycle (Nye, 1964; Steptoe et al., 1976), and it is formally assessed in conjunction with a continuous electrocardiogram (ECG) (Geddes et al., 1981). The PTT indirectly depends on BP, as higher pressure results in a faster PTT (Geddes et al., 1981). While these methodologies could serve as a foundation for the development of similar CO estimation methods, conventional cuffless PTT-based estimation methods are still subject to cuff dependence as they necessitate at least one-time calibration.

A cuff-free, portable device that can measure CO without calibration would be valuable for continuous CO measurement, but it does not currently exist. Our analysis indicated that, in an *in silico* population generated from a numerical model of the cardiovascular system, neural networks could enable the estimation of CO with the elimination of the calibration process, leading to a completely cuff-free solution. Such a solution will ultimately permit the integration of predictive models in wearable technologies. It is important to acknowledge that currently, smartwatches and fitness bands are not optimized to deliver precise and repeatable methods for recording pressure signals. However, the future holds promise (Liao et al., 2019), as sensing technologies continue to advance rapidly.

Undoubtedly, this study relies entirely on *in silico* data which, despite the high complexity of the adopted numerical model and the attentive design of the data generation, correspond to nearly perfect conditions. It is possible that there will be circumstances in real world clinical practice in which the *in silico* results may not be in line with the *in vivo* findings. Nevertheless, this model has undergone comprehensive validation in previous studies using in vivo data. Therefore, it can serve as an excellent starting point for evaluating the concept of cuff-free CO estimation, solely relying on the uncalibrated radial BP waveform as input. As gold-standard CO measurements require the use of invasive catheter-based procedures, it is worth verifying that the initial hypothesis is evaluated and validated *in silico*. Furthermore, we conducted tests on the proposed method while considering artificially introduced errors. This allowed us to assess its performance using data that could better simulate realistic data registries. Positive results, as those produced by this study, can now be the basis for extending the analysis on human data and investigating the validity of this method in the clinic. Of particular interest is to evaluate this method’s accuracy in children’s populations, as childhood hemodynamics differ inherently in comparison to adult hemodynamics [e.g., minimum levels of aortic systolic blood pressure may be equal to 60 mmHg (Zinoveev et al., 2019)]. Furthermore, as a next step, we intend to assess the model’s performance in individuals performing low-, moderate-, or high-intensity physical activity. Yet, based on the sensitivity analysis of the prediction error, we demonstrated that the estimation error is not correlated to the value of mean aortic blood pressure and heart rate. Moreover, future work will include testing the accuracy of the proposed methodology using clinical data from both healthy subjects (controls) and patients with various forms of cardiovascular disease (especially in cases where the presence of extreme CO levels is possible). The latter will allow to evaluate the method’s discrimination capacity and its utility for risk stratification. Lastly, future modifications of the model relying on the uncalibrated radial BP should be performed to overcome a possible systematic error evidenced at higher CO values that are often observed in athletes. This error might be attributed to the limited number of higher CO values in the *in silico* population.

## Conclusion

In this study, we described and validated an ANN-based methodology that allows for the non-invasive estimation of CO from a calibrated or uncalibrated radial BP waveform. This study was motivated by the fact that such a model would be of great value for easy and continuous CO monitoring in everyday life, optimizing patient management. The evaluation of the hypothesis that the uncalibrated radial BP waveform contains sufficient information for accurately deriving CO was found to be true in a large *in silico* population of virtual subjects. We plan to investigate the validity of our results using *in vivo* human data. The latter will verify the clinical utility of the proposed model, while it will enable research applications for the integration of the model in wearable sensing systems, such as smartwatches or other consumer devices.

## Data Availability

The raw data supporting the conclusion of this article will be made available by the authors, without undue reservation.
